# Nonthyroidal Illness Syndrome: To Treat or Not to Treat? Have We Answered the Question? A Review of Metanalyses

**DOI:** 10.3389/fendo.2022.850328

**Published:** 2022-05-10

**Authors:** Salvatore Sciacchitano, Carlo Capalbo, Christian Napoli, Paolo Anibaldi, Valentina Salvati, Claudia De Vitis, Rita Mancini, Flaminia Coluzzi, Monica Rocco

**Affiliations:** ^1^Department of Clinical and Molecular Medicine, Sapienza University of Rome, Rome, Italy; ^2^Laboratory of Biomedical Research, Niccolò Cusano University Foundation, Rome, Italy; ^3^Unit of Medical Oncology, Sant’Andrea University Hospital, Rome, Italy; ^4^Department of Molecular Medicine, Sapienza University of Rome, Rome, Italy; ^5^Department of Surgical and Medical Science and Translational Medicine, Sapienza University of Rome, Rome, Italy; ^6^Health Management Director, Sant’Andrea University Hospital, Rome, Italy; ^7^Scientific Direction, IRCCS Regina Elena National Cancer Institute, Rome, Italy; ^8^Unit of Anesthesia, Intensive Care and Pain Medicine, Sant’Andrea University Hospital, Rome, Italy; ^9^Department Medical and Surgical Sciences and Biotechnologies, Sapienza University of Rome, Polo Pontino, Latina, Italy

**Keywords:** nonthyroidal illness syndrome, SARS-CoV-2 (2019-nCoV) coronavirus, bioelectrical impedance analysis, hydration, sodium/potassium exchangeable ratio

## Abstract

**Background and Objective:**

Nonthyroidal Illness Syndrome (NTIS) occurs in approximately 70% of patients admitted to Intensive Care Units (ICU)s and has been associated with increased risk of death. Whether patients with NTIS should receive treatment with thyroid hormones (TH)s is still debated. Since many interventional randomized clinical trials (IRCT)s were not conclusive, current guidelines do not recommend treatment for these patients. In this review, we analyze the reasons why TH treatment did not furnish convincing results regarding possible beneficial effects in reported IRCTs.

**Methods:**

We performed a review of the metanalyses focused on NTIS in critically ill patients. After a careful selection, we extracted data from four metanalyses, performed in different clinical conditions and diseases. In particular, we analyzed the type of TH supplementation, the route of administration, the dosages and duration of treatment and the outcomes chosen to evaluate the results.

**Results:**

We observed a marked heterogeneity among the IRCTs, in terms of type of TH supplementation, route of administration, dosages and duration of treatment. We also found great variability in the primary outcomes, such as prevention of neurological alterations, reduction of oxygen requirements, restoration of endocrinological and clinical parameters and reduction of mortality.

**Conclusions:**

NTIS is a frequent finding in critical ill patients. Despite several available IRCTs, it is still unclear whether NTIS should be treated or not. New primary endpoints should be identified to adequately validate the efficacy of TH treatment and to obtain a clear answer to the question raised some years ago.

## Introduction

Almost fifteen years ago Robin P. Peeters (1) raised the following question: should we treat Nonthyroidal Illness Syndrome (NTIS) or not? We still do not have a definite answer to this question. In Intensive Care Units (ICU)s, this syndrome occurs rather frequently as a complication of critical diseases affecting virtually all systems and organs. NTIS can be diagnosed in up to 70% of the critically ill patients (2–5) of all ages, including preterm neonates, term infants, children and adults (6). NTIS, observed in ICU in association with all these conditions, has a negative prognostic impact on the course of the disease with a relevant increased risk of death (7–10). Moreover, it represents an independent predictor of short- and long-term survival in patients with myocardial infarction, heart failure, or acute stroke also outside the ICU setting (11). This syndrome occurs frequently in COVID-19 patients too (12). We studied COVID-19 patients during two pandemic waves (13–16). Although it has been reported that patient’s characteristics, such as age, sex, occurrence of comorbidities, symptoms and survival time largely differed during the different phases of the pandemic in Italy (17), we found a similar frequency of ~ 60% of NTIS in our patients during two pandemic waves (18, 19). As reported in other critical illness, NTIS was associated with a more severe disease and a higher rate of lethality (18–20).

Even if patients with NTIS show a clear and marked reduction in the serum FT3 levels and, possibly, in the serum FT4 levels too, there is great uncertainty about the benefit of thyroid hormone (TH) replacement therapy. This could also be related to the fact that the syndrome has “different faces” (21). According to L. De Groot, there are many possible conceptual hypotheses to explain the physiological basis of NTIS (22), including TH test artifacts, inhibitors of T4 binding to proteins or to nuclear T3 receptors and enhanced local deiodination activity. However, the question whether the NTIS represents a beneficial, protective and appropriate adaptive response to stress, critical illness and malnutrition or it should be considered a maladaptive response to illness that requires correction still remains unanswered (23). We still do not have convincing data regarding any potential benefit due to TH treatment. Therefore, treatment with TH is not recommended in the absence of clinical signs of hypothyroidism (24). Although the administration of low doses of TH has not been linked to any harm (25–28), there is lack of convincing evidences regarding any clinical benefit (29). One of the reasons could be due to the use of inadequate outcome indicators.

We reviewed the data reported in metanalyses, regarding the interventional randomized clinical trials (IRCT)s aimed to demonstrate the benefit of the treatment with THs in NTIS associated with different conditions and diseases. Conflicting results obtained indicate that more adequate and pathogenic primary outcomes are needed. The possible use of new better endpoints, such as the hydration parameters measured by Bioelectrical Impedance Analysis (BIA) is discussed.

## Methods

### Literature Search and Study Selection

**Figure 1:**
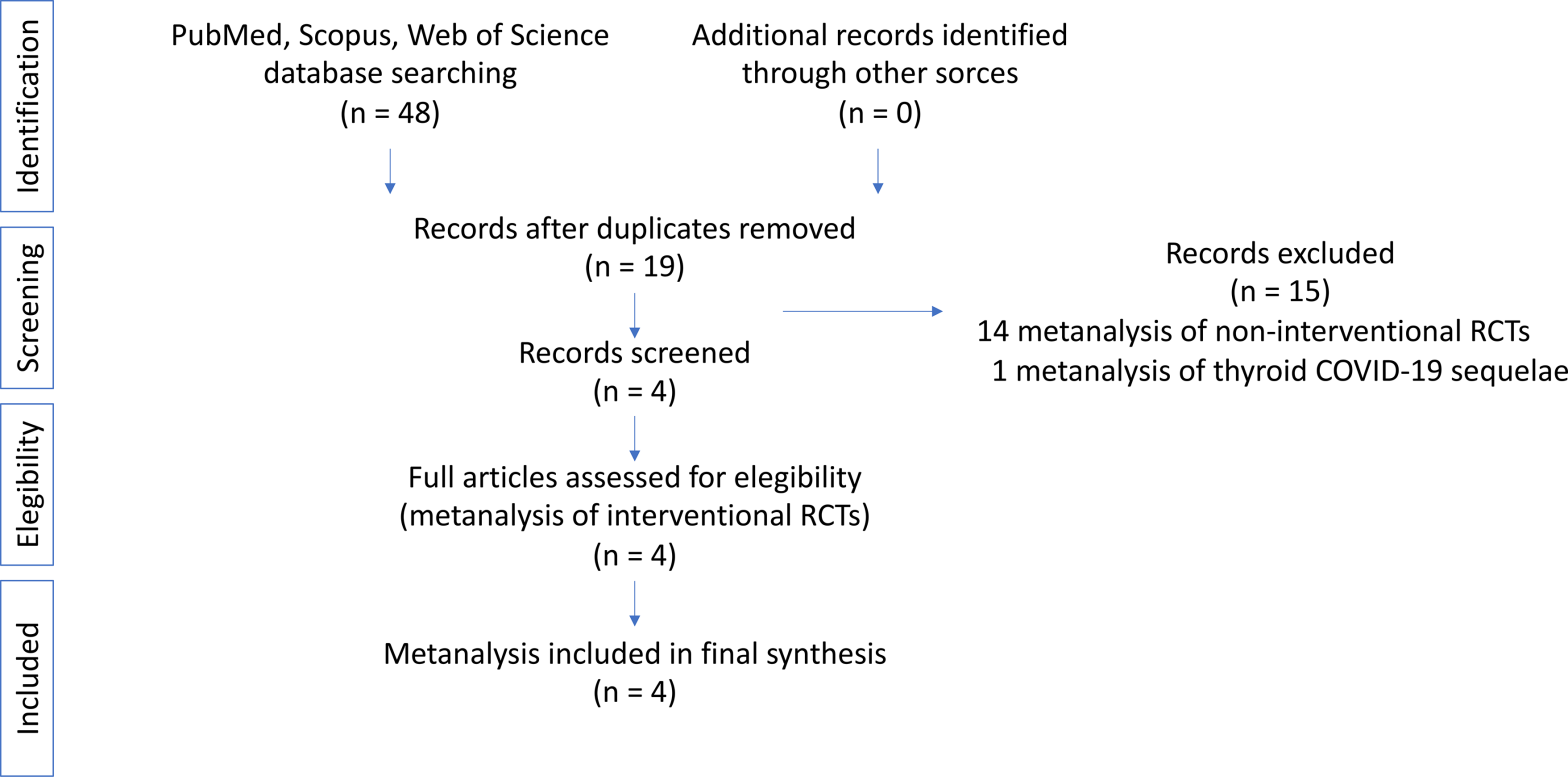
Search strategy and flow of metanalyses.

We analyzed the existing published high-quality metanalyses regarding IRCTs performed to treat critical ill patients with NTIS by administrating TH supplementation. For this reason, we undertook a search *via* PubMed, Scopus and ISI Web of Knowledge until 1^st^ December 2021. The search terms were: “meta-analysis” OR “meta-analyses” AND “Nonthyroidal Illness Syndrome (NTIS)”, “Euthyroid Sick Syndrome (ESS)”, Low T3 Syndrome (LT3S)”. The review was conducted following the guidance proposed by Aromataris et al. (30). Due to the specific goals of this systematic review, specifically focused on metanalyses of IRCTs, data regarding the frequency of NTIS or its role as prognostic factor were excluded. The search strategy and flow of the metanalyses is illustrated in **Figure 1**. Two authors (FC and SS) carefully reviewed every original article. After this process, another author (CC) repeated the analysis to ensure reliability.

### Data Items Collection Process

Having identified the final list of metanalyses, each article contained within each metanalysis was thoroughly reviewed in light of the following questions: 1) which disease/condition was considered; 2) how many patients were included in the metanalysis; 3) what was the type and dosage of THs supplementation; 4) how long was the duration of treatment; 5) what was the primary outcome measured; and 6) did the treatment produce any clinical benefit with respect to the primary outcome.

### Assessment of the Quality of the Selected Metanalyses

We used the MeaSurement Tool to Assess systematic Reviews 2 (AMSTAR 2) to evaluate the methodological quality of the studies included in the selected metanalyses (31). We calculated the AMSTAR score by summing up all yes answers (yes = 1). The reviews were then categorized with respect to their methodological quality and based on their AMSTAR score. Categories of quality were determined as follows: low (score 0 to 4), medium (score 5 to 8), and high (score 9 to 11). The quality of the included metanalyses was assessed by two researchers independently to confirm accuracy of the analysis. Discrepancies were resolved by consensus.

## Results

### Results of the Literature Search

A total of 48 metanalyses emerged from the initial search process of the literature. Four of them remained after manual removal of duplicates and after a screening process. Fourteen studies were excluded because they refer to non-IRCTs and one because was focused on thyroid sequelae of COVID-19. The selected metanalyses were assessed for eligibility and deemed appropriate for inclusion. A flow diagram representing this search strategy is presented in **Figure 1**. As a result of the extensive literature search and screening of metanalyses concerning IRCTs in NTIS, we found 4 metanalyses matching our selection criteria. The data extracted from each metanalysis are reported in **Table 1**.

**Table 1:** Selected metanalyses of IRCTs in NTIS.

Meta-analyses (year) [ref]	Conditions Disease	N. of IRCTs included,Author, year	N. of patients Study group(Placebo group)	Intervention	Primary Outcome Measures	Beneficial effects
Osborn D.A. (2007) (32)	Preterm infants	**4** Smith et al. (36) Valerio et al. (37) van Wassenaer et al. (38) Vanhole et al. (39)	**170 (148)** 29 (18) 21 (10) 100 (100) 20 (20)	LT4 8-20 μg/kg/day or LT3 0.5 μg/kg → LT4 8-20 μg/kg/day	Neurodevelopment, Oxygen need, Endocrine/clinical outcomes, Mortality	No evidence that THs, routinely administered in preterm babies, are effective in preventing developmental problems
Kaptein E.M. (2010) (33)	Cardiac Surgery (13) kidney transplant (1)	**14** Klemperer et al. (25) Bennett-Guerrero et al. (26) Klemperer et al. (40) Mullis-Jansson et al. (41) Guden et al. (42) Ranasinghe et al. (43) Acker et al. (44) Novitzky et al. (45) Teiger et al. (46) Vavouranakis et al. (47) Spratt et al. (48) Sirlak et al. (49) Magalhaes et al. (50) Choi et al. (51)	**538 (643)** 71 (71) 66 (71) 66 (65) 81 (89) 30 (30) 63 (160) 20 (18) 13 (11) 10 (10) 15 (15) 30 (28) 40 (40) 8 (10) 25 (25)	LT3 iv (high doses) 0.175-0.333 μg/kg/h, for 6-9 h LT3 iv (low doses) 0.0275-0.0333 μg/kg/h, for 14-24 h LT3 orally variable doses and durations	Cardiac index Systemic vascular resistance Heart rate Atrial Fibrillation Inotrope use Mortality	TH treatment increases: Cardiac index No evidence that TH treatment is effective on mortality
Liu X. (2014) (34)	Nephrotic Syndrome	**6** Duan et al. (52) Hu et al. (53) Li et al. (54) Nie et al. (55) Zhang et al. (56) Xie et al. (57)	**174 (156)** 40 (38) 26 (24) 25 (23) 36 (30) 26 (24) 20 (17)	LT4 20-50 μg/kg/day	Remission of nephrotic syndrome	No side effect was observed TH treatment increases remission of nephrotic syndrome
Flores S. (2019) (35)	Congenital Heart Surgery	**9** Bettendorf et al. (58) Chowdhury et al. (59) Mackie et al. (60) Mainwaring et al. (61) Marwali et al. (62) Marwali et al. (63) Portman et al. (64) Portman et al. (65) Talwar et al. (66)	**364 (347)** 20 (20) 14 (14) 22 (20) 21 (7) 28 (30) 104 (104) 7 (7) 98 (95) 50 (50)	LT3 iv 0.4-1.2 μg/kg/h LT3 enteral 0.5-5.0 μg/kg/h	Cardiac index MV duration ICU stay duration Post-operative hospital stay duration Inotrope score Mortality	No evidence that TH treatment is effective on: MV duration, Hospital stay duration, Cardiac index, Mortality TH treatment ameliorates: Inotrope score, ICU stay duration
**Total**		**33**	**1,246 (1,294)**			

CLD, chronic lung disease; CPB, cardiopulmonary bypass; ICU, intensive care unit; i.v., intravenous; MV, mechanical ventilation; NS, nephrotic syndrome; IRCTs, interventional randomized controlled trials; ROA, route of administration; TH, thyroid hormone; LT3, lio-triiodothyroinine; LT4, levothyroxine.

For the reference of each single RCT reported in the table see the specific metanalysis where it is included. Bold numbers refer to the total number of studies (column #3) and patients (column #4).

### Quality of the Selected Metanalyses

The results of the evaluation of the quality of the metanalyses included in the review are reported (**Supplementary Table 1**). The four metanalyses selected showed a medium/high score, according to the 16 points included in AMSTAR 2 check list. However, it should be considered that although the metanalysis on nephrotic syndrome by Liu et al. was based on IRCTs from China, which were written in Chinese and have not been published in peer-reviewed journals available on PubMed, the quality was high according to the AMSTAR 2 score.

### Type of Conditions/Diseases

The four metanalyses were focused on NTIS associated with various diseases and conditions. One of them evaluated TH replacement efficacy in 14 IRTCs performed in adult patients who underwent to cardiac surgery (33). In another one the same evaluation was conducted in 9 IRCTs, performed in children after congenital heart surgery (35). The potential benefit of TH replacement was evaluated in a metanalysis focused on 6 IRCTs, performed in patients with nephrotic syndrome (34). Finally, in another metanalysis (32), the potential effect of prophylactic postnatal TH supplementation in preventing morbidity and mortality was evaluated in 4 IRCTs performed in preterm infants. The total number of IRCTs, included in the four metanalyses was 33, with a total of 1,245 patients enrolled in the study group and received treatment with either LT4 or LT3. Results were compared to a total of 1,294 patients enrolled in the control group, which received placebo or no treatment.

### Types of Interventions and Dosage Strategy

The IRCTs included in the 4 metanalyses showed marked differences in the type of TH supplementation, in the route of administration as well as in the dosages and duration of treatment. In the 4 IRCTs included in the metanalysis reported by Osborn et al. (32), treatment of preterm infants (25-31 weeks’ gestation) consisted in the administration of intravenous (iv) LT4 until tolerating feeds, then orally, starting from 12 - 24 hours of age, at doses ranging from 8 to 20 μg/kg daily for up to six weeks. In some IRCTs, before administration of LT4 a pretreatment with LT3 at 0.5 μg/kg/h was started 24 hours after birth. Treatment with LT4, at doses ranging from 20 to 50 μg/kg/day, was also the treatment of choice given to patients with nephrotic syndrome in the 6 IRCTs included in the metanalysis reported by Liu et al. (34). In the 14 IRCTs included in the metanalysis by Kaptein et al. (33), patients received treatment with iv LT3 at high doses (0.175-0.333 μg/kg/h) or at low doses (0.0275-0.0333 μg/kg/h) or orally at variable doses. Finally, in the eight IRCTs included in the metanalysis by Flores et al. (35), patients that underwent to cardiac surgery were treated post-operatively with LT3, given intravenously or enterally, at doses ranging from 0.4 to 5.0 μg/kg/h.

### Outcomes

Since the disease associated with NTIS were different and affected several apparatuses, the outcomes were extremely variable too, reflecting the aim of each IRCT to demonstrate improvement in the function of the specific organ/system involved. In IRCTs designed to evaluate the efficacy of prophylactic LT3 treatment in preterm infants, the chosen outcomes were the following: prevention of neurological alterations, reduction of oxygen requirements, restoration of endocrinological and clinical parameters and reduction of mortality. In adult patients that underwent to cardiac surgery, the efficacy of TH supplementation was assessed based on the effects on cardiovascular parameters, such as the cardiac index, the systemic vascular resistance, the heart rate, the occurrence of atrial fibrillation, the use of inotropes and, finally, mortality. The primary outcome in patient with nephrotic syndrome was the remission of the disease. In pediatric patients that were surgically treated for cardiac congenital heart defects, the outcomes were mostly related to cardiac function and consisted in the cardiac index, the inotrope score, the duration of mechanical ventilation (MV), the length of stay in ICU and in the hospital after operation, and mortality.

### Effects of Interventions With THs on the Chosen Outcomes

Analysis of the results reported by the metanalyses and concerning the effects of LT3 in both pediatric and adult patients that underwent cardiac surgery are inconclusive. An effect of LT3 on cardiac index has been reported in adult patients but not in pediatric ones. No clear effects on mortality were reported in all IRCTs, performed both in children and adult patients. In children that were surgically treated for congenital heart defects, LT3 supplementation did not significantly alter also the postoperative course of the disease in any of the parameters considered, namely duration of mechanical ventilation, duration of postoperative hospital stays, cardiac index at 24 hours postoperatively, and inpatient mortality. A limited effect was reported in the inotrope score and in the duration of ICU stay. In one IRCT, perioperative LT3 administration decreased the incidence and need for antiarrhythmic treatment of postoperative atrial fibrillation (40). Better results have been reported in the IRCTs performed in nephrotic patients, where LT4 treatment produced a significant increase in the total remission rate of the disease. Analysis of the results reported in the IRCTs performed in preterm infants showed no evidence of any beneficial effects of the prophylactic administration of LT4, with respect of neurodevelopment, oxygen need, restoration of endocrine and clinical parameters and on mortality. Heterogeneity of the diseases considered in each RCT and of the therapeutic approaches used as well as of the measures chosen to evaluate the potential beneficial effects make difficult to draw any general conclusion.

## Discussion

### Metanalyses of IRCTs With THs in Patients NTIS

There have been many attempts in the past to demonstrate the benefit of treatment with THs in NTIS. Treatment with LT4 proved to be not beneficial (67) and treatment with LT3 was ineffective too (68) or showed appreciable improvement (69–73). Heart failure, cardiac surgery and acute myocardial infarction appear to be interesting areas of investigation. NTIS is frequently detected in these patients and many trials have analyzed the possible favorable effects of either LT4 or LT3. Some results have been obtained by treating with LT3 patients with cardiac diseases or those undergoing cardiothoracic or coronary bypass procedures (25, 27, 45, 74–77). However, after an initial enthusiasm the results of the trials were disappointing (46). In 2 metanalyses of IRCTs performed in both pediatric and adult patients that underwent to cardiac surgery, treatment with LT3, administered either intravenously at low or high doses or enterally, proved to have beneficial effects only with regard to some endpoints, namely the cardiac index, the use on antiarrhythmic drug for atrial fibrillation and the inotrope score, but it was not effective in two major outcomes, namely mortality and duration of mechanical ventilation. Since THs are widely recognized as potent stimulators of fetal lung maturation and of surfactant production in the newborns, the use of TH supplementation has, therefore, been suggested in preterm infants. We retrieved 1 metanalysis, including 4 IRCTs in which the potential benefit of LT4 was assessed with respect to two outcomes i.e., oxygen requirement and mortality. In addition, the occurrence of late sequelae, consisting in endocrinological and neurodevelopmental alterations, were also analyzed. The 4 IRCTs considered in this metanalysis, failed to demonstrate any beneficial effects of prophylactic LT4 treatment in these subjects. Conversely, the administration of LT4 proved to be effective in inducing the remission of the NTIS and of the nephrotic syndrome, indicating a therapeutic effect of THs on hydroelectrolytic homeostasis and on renal hemodynamics.

However, after many years and many published papers as well as many IRCTs, the question whether NTIS should be considered an euthyroid condition that doesn’t require treatment or a truly hypothyroid disorder that may benefit from treatment with thyroid hormone remains unanswered. We are still waiting for additional IRCTs, based on adequate doses of THs and proper time frame to evaluate the effects of treatment and, most important, based on more appropriate primary outcome measures and on valid pathogenic biomarkers to assess the potential benefit.

There are several possible reasons why many of the IRCTs failed: i) the treatment was not appropriate and LT4 was used instead of LT3 or a combination of both; ii) the treatment was given at dosage unappropriated; or iii) the chosen primary endpoint was not adequate to evaluate the efficacy of the treatment. The last point is crucial. In many cases, in fact, it is difficult to find congruence of the conclusions regarding the effects of the treatments and there is lack of evidence regarding a strong pathophysiological mechanism of action or the final target of the hormone. Selection of non-appropriate endpoints could result in failure of the trial in achieving statistical significance (78). Decision whether to treat or not to treat should be based on evidences obtained using the best possible and hard clinical endpoint and not on surrogates (79, 80). The availability of a suitable and clear gold-standard endpoint would certainly facilitate the analysis of the benefit of TH treatment in future IRCTs in patients with NTIS.

### Future Perspectives

Our review does make it apparent that it is difficult to draw any conclusions and this represents an area that remains to be explored. New adequate outcomes are needed to evaluate potential beneficial effects of THs in NTIS. During the last COVID-19 pandemic wave, we analyzed critical ill patients, admitted to the ICU of our Hospital, by means of BIA and we demonstrated that low FT3 serum values were associated with profound changes in the hydroelectrolytic equilibrium at the periphery, responsible for marked water and salt retention and generalized edema (18). The development of NTIS occurred acutely in these patients. However, the picture was similar to that observed in myxedema due to chronic, longstanding and progressive hypothyroidism. We observed an increase in the total body water (TBW) as well as in the fraction of free fat mass (FFM) as water, expressed as the TBW/FFM ratio, also indicated as hydration, in COVID-19 patients with NTIS, compared to those with normal FT3 serum values. Such changes were associated with an increase in the Na*_e_* : K*_e_* exchangeable ratio, always measured by BIA and with a reduced mRNA expression levels of the two genes coding for the two major subunits of the Na+/K+ pump, in the PBMCs obtained from COVID-19 patients during the acute phase of the disease (18).

These results suggest that the primary change in NTIS could possibly be the reduced expression/activity of the Na^+^/K^+^ pump. It is well known that THs are key determinants of cellular metabolism in many target tissues (81). The Na^+^/K^+^ pump has been known for a long time as a target of T3 transcriptional as well as non-transcriptional activity (82–85). In cultured chick cardiac myocytes, inhibition of Na^+^/K^+^ pump promotes the efflux of ions and water, causing cell shrinkage (86). Na^+^ efflux is an energy consuming process that has been attributed to the ATP-dependent Na^+^/K^+^ pump. Activation of this pump leads to the exit of 3 Na^+^ ions for every 2 K^+^ ions entering the cell. According to the pump-leak hypothesis (87, 88), inhibition of the Na^+^/K^+^ pump activity should result in cell swelling. However, it has been demonstrated that the effect of Na^+^/K^+^ pump inhibition is, indeed, not swelling, but apoptotic cell shrinkage (86, 89). The regulation, mechanism of action as well as the role of Na^+^/K^+^ pump in many human diseases, however, is not fully understood yet (90). We may speculate that Na^+^/K^+^ pump downregulation, due to an acute decrease in LT3 serum concentration, would lead to an efflux of ions and other molecules outside the cellular membrane. Water would follow as a consequence of osmotic attraction. The LT3-mediated downregulation of the Na^+^/K^+^ pump could be the likely pathogenic mechanisms, responsible for the cellular damage observed in NTIS.

## Conclusion

We performed an extensive search in the literature, looking for metanalyses of IRCTs performed in NTIS affecting critically ill patients in the ICU setting. We investigated the reasons why several clinical trials failed and why there is still lacking of convincing data regarding the efficacy of TH supplementation in patients with NTIS. We found extreme variability in terms of conditions or diseases associated with NTIS, types, dosages and routes of administration and duration of TH supplementation used in the various IRCTs. This may reflect the peculiarity of NTIS, considered a “syndrome with many different faces”. In particular, we found that in each IRCT included in the selected metanalysis the chosen primary outcomes seem to be not appropriate or not accurately defined for all the pathological conditions associated with NTIS. Our experience in COVID-19 patients with NTIS indicates that BIA parameters, and in particular hydration and Na*_e_* : K*_e_* ratio, are suitable markers for the assessment of the hydroelectrolytic balance at the periphery in NTIS and may represent adequate clinical endpoints to evaluate the efficacy of TH treatment in future IRCTs. We may, therefore, be close to yield a definite answer to the question concerning the potential benefit of treatment with LT3 in critically ill patients with NTIS.

## Author Contributions

SS principal investigator. SS, CN, and PA designed and supervised the review. CV and VS performed the literature search. SS, CC, and FC carefully reviewed every article. SS and FC performed the analysis of the quality through the AMSTAR 2 score system. SS, FC, RM, and MR wrote the manuscript. All authors contributed to the article and approved the submitted version.

## Funding

This work was supported by: (1) Italian Association for Cancer Research (AIRC) grants IG24451 to RM, (2) the LazioInnova grant 2018 n.85-2017-13750 to RM, (3) the Sapienza University Research Projects of National Relevance - PRIN 2017 (Prot. 2017HWTP2K) to RM. The funders had no role in study design, data collection and analysis, decision to publish, or preparation of the manuscript.

## Conflict of Interest

The authors declare that the research was conducted in the absence of any commercial or financial relationships that could be construed as a potential conflict of interest.

## Publisher’s Note

All claims expressed in this article are solely those of the authors and do not necessarily represent those of their affiliated organizations, or those of the publisher, the editors and the reviewers. Any product that may be evaluated in this article, or claim that may be made by its manufacturer, is not guaranteed or endorsed by the publisher.

## Abbreviations

TH, thyroid hormone; T3, 3,5,3′-triiodothyronine; T4, thyroxine; LT3, levo-triiodothyronine; LT4, levothyroxine; FT3, free triiodothyronine; FT4, free thyroxine; NTIS, nonthyroidal illness syndrome; ESS, euthyroid sick syndrome; LT3S, low T3 syndrome; IRCT, interventional randomized clinical trial; MV, mechanical ventilation; BIA, bioelectrical impedance analysis; Rz, resistance; Xc, reactance; PhA, phase angle; Na*_e_* : K*_e_*, sodium/potassium exchangeable ratio; BCM, body cell mass; BMI, body mass index; TBW, total body water; ECW, extra cellular water; ICW, intra cellular water; FFM, fat-free mass; FM, fat mass; PBMCs, peripheral blood mononuclear cells.
