# Supplementary material for: Nonthyroidal Illness Syndrome: To Treat or Not to Treat? Have We Answered the Question? A Review of Metanalyses
Source: Front Endocrinol (Lausanne). 2022 May 10;13:850328. doi: 10.3389/fendo.2022.850328 (PMC9128382; doi:10.3389/fendo.2022.850328)
Supplement: Supplementary file 1 [file Table_1.docx]

**Suppl. Table 1: Quality of the selected metanalyses of IRCTs in NTIS**

| **Meta-analyses (year) [ref]** | **Conditions Disease** | **Question number^a^** | | | | | | | | | | | | | | | | **AMSTAR 2 Score** | **Overall confidence** |
| --- | --- | --- | --- | --- | --- | --- | --- | --- | --- | --- | --- | --- | --- | --- | --- | --- | --- | --- | --- |
|  |  | **1** | **2** | **3** | **4** | **5** | **6** | **7** | **8** | **9** | **10** | **11** | **12** | **13** | **14** | **15** | **16** |  |  |
| Osborn D.A. (2007) [35] | Preterm infants | no | yes | yes | yes | no | no | yes | yes | yes | no | no | no | yes | yes | no | no | 8 | medium |
| Kaptein E.M. (2010) [32] | Cardiac Surgery  Kidney transplant | no | yes | yes | yes | yes | yes | yes | yes | no | no | yes | yes | yes | yes | no | no | 11 | high |
| Liu X.  (2014) [34] | Nephrotic Syndrome | no | yes | yes | yes | yes | yes | yes | yes | yes | no | yes | yes | yes | yes | no | no | 12 | high |
| Flores S. (2019) [33] | Congenital Heart Surgery | no | yes | yes | yes | yes | yes | no | yes | yes | no | yes | yes | no | no | no | no | 9 | high |

IRCTs: interventional randomized clinical trials; NTIS: Nonthyroidal Illness Syndrome

^a^The questions are those of the MeaSurement Tool to Assess systematic Reviews 2 (AMSTAR 2) (Shea BJ, Reeves BC, Wells G, Thuku M, Hamel C, Moran J, Moher D, Tugwell P, Welch V, Kristjansson E, Henry DA. AMSTAR 2: a critical appraisal tool for systematic reviews that include randomised or non-randomised studies of healthcare interventions, or both. BMJ. 2017 Sep 21;358:j4008)

#1. Did the research questions and inclusion criteria for the review include the components of PICO?
#2. Did the report of the review contain an explicit statement that the review methods were established prior to conduct of the review and did the report justify any significant deviations from the protocol?

#3. Did the review authors explain their selection of the study designs for inclusion in the review?

#4. Did the review authors use a comprehensive literature search strategy?
#5. Did the review authors perform study selection in duplicate?
#6. Did the review authors perform data extraction in duplicate?

#7. Did the review authors provide a list of excluded studies and justify the exclusions?
#8. Did the review authors describe the included studies in adequate detail?
#9. Did the review authors use a satisfactory technique for assessing the risk of bias in individual studies that were included in the review?
#10. Did the review authors report on the sources of funding for the included studies?
#11. If meta-analysis was performed, did the review authors use appropriate methods for statistical combination of results?
#12. If meta-analysis was performed, did the review authors assess the potential impact of risk of bias in individual studies on the results of the meta-analysis of other evidence synthesis?
#13. Did the review authors account for risk of bias in individual studies when interpreting/discussing the results of the review?
#14. Did the review authors provide a satisfactory explanation for, and discussion of, any heterogeneity observed in the results of the review?
#15. If they performed quantitative synthesis did the review authors carry out an adequate investigation of publication bias (small study bias) and discuss its likely impact on the results of the review?

#16. Did the review authors report any potential sources of conflict of interest, including any funding they received for conducting the review?

CD, cannot determine; NA, not applicable.
